# Role of *ZBTB38* Genotype and Expression in Growth and Response to Recombinant Human Growth Hormone Treatment

**DOI:** 10.1210/jendso/bvac006

**Published:** 2022-01-18

**Authors:** Samuel Parsons, Adam Stevens, Andrew Whatmore, Peter E Clayton, Philip G Murray

**Affiliations:** 1 Division of Developmental Biology and Medicine, Faculty of Biology, Medicine and Health, University of Manchester and Manchester Academic Health Science Centre, Manchester M13 9WL, UK; 2 Department of Paediatric Endocrinology, Royal Manchester Children’s Hospital, Manchester M13 9WL, UK

**Keywords:** ZBTB38, height, short stature

## Abstract

**Context:**

Single-nucleotide polymorphisms (SNPs) in *ZBTB38* have been associated with idiopathic short stature (ISS) and adult height.

**Objective:**

This study sought to (a) characterize the phenotype of ISS patients and their response to recombinant human growth hormone (rhGH) by *ZBTB38* SNP genotype; (b) describe the relationship of *ZBTB38* expression with normal growth; and (c) describe the in vitro effects of ZBTB38 knockdown on cell proliferation and *MCM10* expression.

**Methods:**

The genotype-phenotype relationship of rs6764769 and rs724016 were explored in 261 ISS patients and effects of genotype on response to rhGH were assessed in 93 patients treated with rhGH. The relationship between age and *ZBTB38* expression was assessed in 87 normal children and young adults. Knockdown of *ZBTB38* in SiHA cells was achieved with siRNAs and cell proliferation assessed with a WST-8 assay.

**Results:**

We found that rs6764769 and rs724016 are in linkage disequilibrium. The rs724016 GG genotype was associated with lower birth length (*P* = 0.01) and a lower change in height SDS over the first year of treatment (*P* = 0.02). *ZBTB38* expression was positively correlated with age (*P* < 0.001). siRNA-mediated knockdown of *ZBTB38* resulted in increased cell proliferation at 72 and 96 hours posttransfection but did not alter expression of *MCM10*.

**Conclusions:**

SNPs within *ZBTB38* associated with ISS are linked to higher birth size within a cohort of ISS patients and a better response to rhGH therapy while *ZBTB38* expression is positively related to age.

Short stature remains one of the commonest reasons for consulting a pediatric endocrinologist, with clinical and biochemical assessment reaching an organic diagnosis in around 15% of short children [1]. The remainder are left with diagnoses based on a description of their growth pattern—familial short stature, ISS, small for gestational age, and constitutional delay of growth. Recent advances in genetic technology allow a molecular diagnosis of a monogenic growth disorder to be made in around another 15% of short children [2] but this still leaves ~70% of children without an endocrine or molecular diagnosis.

Twin studies suggest that the genetic heritability of human height is around 80% to 90% [3], and to date genome-wide association studies (GWAS) studies have explained 24.6% of the variation in human height [4]. It is likely that a proportion of the remaining patients with unexplained short stature will have an undiagnosed monogenic disorder, but others will have a polygenic short stature with the inheritance of many separate genetic variants each producing a small reduction in height.

One gene potentially contributing to polygenic short stature is Zinc-finger and BTB Domain Containing 38 (*ZBTB38*). A total of 13 single-nucleotide polymorphisms (SNPs) either within or in close proximity to *ZBTB38* have been associated with adult height in 9 GWAS studies, making this one of the commonest recurring loci associated with adult height [5-13]. For one of the SNPs, rs6763931 (an intronic variant), Gudbjartsson identified a relationship with *ZBTB38* expression in blood and adipose tissue [6]. In addition, 3 studies have linked SNPs within *ZBTB38* to idiopathic short stature (ISS). Kim et al and Wang et al both identified 2 missense SNPs in *ZBTB38*, rs62282002 and rs16851435, to be associated with ISS [13, 14]. In the EPIGROW study Clayton et al associated 2 *ZBTB38* SNPs, rs6764769 and rs724016, with ISS [15]; one of these is within the 5′ untranslated region and the other intronic. There is therefore evidence that variants affecting transcription of *ZBTB38* may affect human height and contribute to short stature.

*ZBTB38* encodes for a 1195–amino acid transcription factor that binds methylated DNA [16]. *Cibz*, the mouse homolog of *ZBTB38*, is highly expressed in the brain while *ZBTB38* has much more widespread expression [17]. *Cibz* knockout in mouse myoblast cells lines induced cell death [18] and in mouse embryonic stem cells *Cibz* knockdown inhibited cell proliferation [19]. Fillon et al [16] demonstrated that ZBTB38 binds to methylated DNA at the H19/IGF2 differentially methylated region—a region linked to Silver-Russell Syndrome and Beckwith-Wiedemann syndrome. Miotto et al identified a pathway involving Retinoblastoma binding protein 6 (RBBP6) and minichromosome maintenance protein 10 (MCM10) with ZBTB38 [20]. They proposed a model where RBBP6 is responsible for ubiquitinating and degrading ZBTB38, while ZBTB38 exerts an inhibitory effect on the transcription of MCM10, a DNA replication factor. In yeast, a loss-of-function mutation in the *MCM10* homolog abolishes cell proliferation. More recently, downregulation of *ZBTB38* has been identified to increase expression of the growth regulator *CDKN1C* [21]. Mutations in *CDKN1C* causes the short stature condition IMAGe syndrome as well as being a rare cause of Silver-Russell Syndrome [22].

This study aims to further elucidate the role of ZBTB38 in short stature with a combined clinical and molecular approach exploring: (a) the clinical phenotype associated with *ZBTB38* SNPs; (b) *ZBTB38* expression throughout human growth; (c) effects of *ZBTB38* SNPs on response to recombinant human growth hormone treatment; and (d) in vitro studies of *ZBTB38* knockdown.

## Methods

### Age-Related Expression of *ZBTB38*

Gene expression analysis was conducted on a library of gene expression datasets from normal children with age annotation collated from the NCBI Gene expression Omnibus and EBI ArrayExpress databases as previously described [23]. This cohort included 87 healthy control subjects (44 female and 43 male); 33 were aged 0-4 years, 21 were >4-8 years of age, 12 aged >8-12 years, 16 aged >12-17 years, and 5 aged > 17 years.

### Clinical Data

#### EPIGROW cohort

The EPIGROW database, which has previously reported, was made up of genetic and clinical data from 263 children with short stature without a defined etiology [15]. The cohort was divided according to *ZBTB38* SNP (rs6764769 and rs724016) genotype. Access 2010 (Microsoft, WA, USA) was used to select patients who had their genotype and clinical data recorded. Data were exported via Microsoft Excel version 11 (Microsoft, WA, USA) to SPSS version 22 (IBM, NY, USA). Continuous data was analyzed using a one-way ANOVA and Bonferroni post hoc analysis used to account for multiple comparisons. The categorical data was analyzed using either Fishers exact test or Chi-squared test with Yates correction. A *P* value of < 0.05 was used to determine statistical significance.

#### Growth study cohort

The growth study cohort was made up of clinical data from 97 children treated with growth hormone therapy over a 2-year period in a single center. Informed consent had previously been taken from patients to encompass growth related genetic testing and approval given by the local research ethics committee. Patients with clinical data and available DNA were selected from the cohort.

### Rs6764769 and rs724016 Sequencing

Genomic DNA was isolated from whole blood samples via QIAGEN DNeasy kit (Qiagen, Manchester, UK). Primers were designed to amplify DNA fragments containing each SNP were as follow: rs6764769: Forward, TCAGTTGCTAAAGCCGAGGT; Reverse, AAGCCTTGTGGACCAAACTG and rs724016: Forward, CATCCCGGACCAGATACATA; Reverse, GCAAGATGAGCCCAATCACT. Polymerase chain reaction (PCR) was undertaken using standard laboratory procedures. PCR products were purified with exonuclease I (ExoSAPIT; Amersham Bioscience, Buckinghamshire, UK) according to the manufacturer’s instructions and products were sequenced using Applied Biosystems BDv3.1 on an ABI3730 automated analyzer (Applied Biosystems, Loughbourough, UK) followed by mutation detection using sequence analysis software (Applied Biosystems, Loughbourough, UK).

### Routine Cell Culture

SiHA cells were cultured in 75 cm^2^ cell culture flasks (Corning, Tewkesbury, MA, USA) in DMEM (Invitrogen Paisley, UK) supplemented to a final concentration with 10% fetal bovine serum (Invitrogen), 50 units/mL penicillin, 50 μg/mL streptomycin, 2 mM glutamine and 2.5 μg/mL amphotericin B (Invitrogen, Paisley, UK).

### Small Interfering RNA Knockdown of ZBTB38

All small interfering RNAs (siRNAs) were supplied by Invitrogen (Paisley, UK). Two siRNAs were used to knock down *ZBTB38* expression (assay IDs s48414 and s48415). For each experiment, a nonsilencing siRNA control was included (assay ID 4390844) and a GAPDH-positive control (assay ID 4390849). All siRNAs were transfected at a final concentration of 5nM. Cells were passaged 24 hours prior to siRNA transfection to achieve a cell confluence of 60% and logarithmic phase of growth. Transfection was performed as per the manufacturer’s instructions for the reverse transfection protocol with Lipofectamine 2000 (Invitrogen, Paisley, UK) and Opti-MEM (Invitrogen, Paisley, UK). Cells were seeded into 96-well plates with 7500 cells per well.

### RNA Extraction and Quantitative PCR

The TaqMan cells-to-CT kit (Invitrogen, Paisley, UK) was used to extract RNA and produce cDNA. TaqMan probes (Invitrogen, Paisley, UK) were used for assessment of *ZBTB38* (assay ID Hs0153164_m1) and *MCM10* (assay ID Hs00960349_m1) expression, and *PPIA* expression was determined using standard SYBR green quantitative PCR (primer sequences available upon request). Stratagene Mx3000p/Mx3005P (Life Technologies, Paisley, UK) thermal cyclers were used to determine relative quantitative gene expression which was calculated as 2^-ΔΔCT^.

### Cell Proliferation Assay

Cells were transfected and seeded as per above with nonsilencing siRNA or ZBTB38 siRNA. At 24, 48, 72, and 96 hours after seeding, 12 μL WST-8 (Sigma-Aldrich Company, Poole, Dorset, UK) was added to each well, the plate was incubated for 2 hours at 37 °C before measuring absorbance at 450 nm on a UV spectrophotometer (Bio-Rad Benchmark microplate reader, Bio-Rad UK). For each cell line at each time measurement, a minimum of 3 independent wells were examined on 3 separate occasions.

## Results

### Age-Related Expression of *ZBTB38*

**Figure 1.**
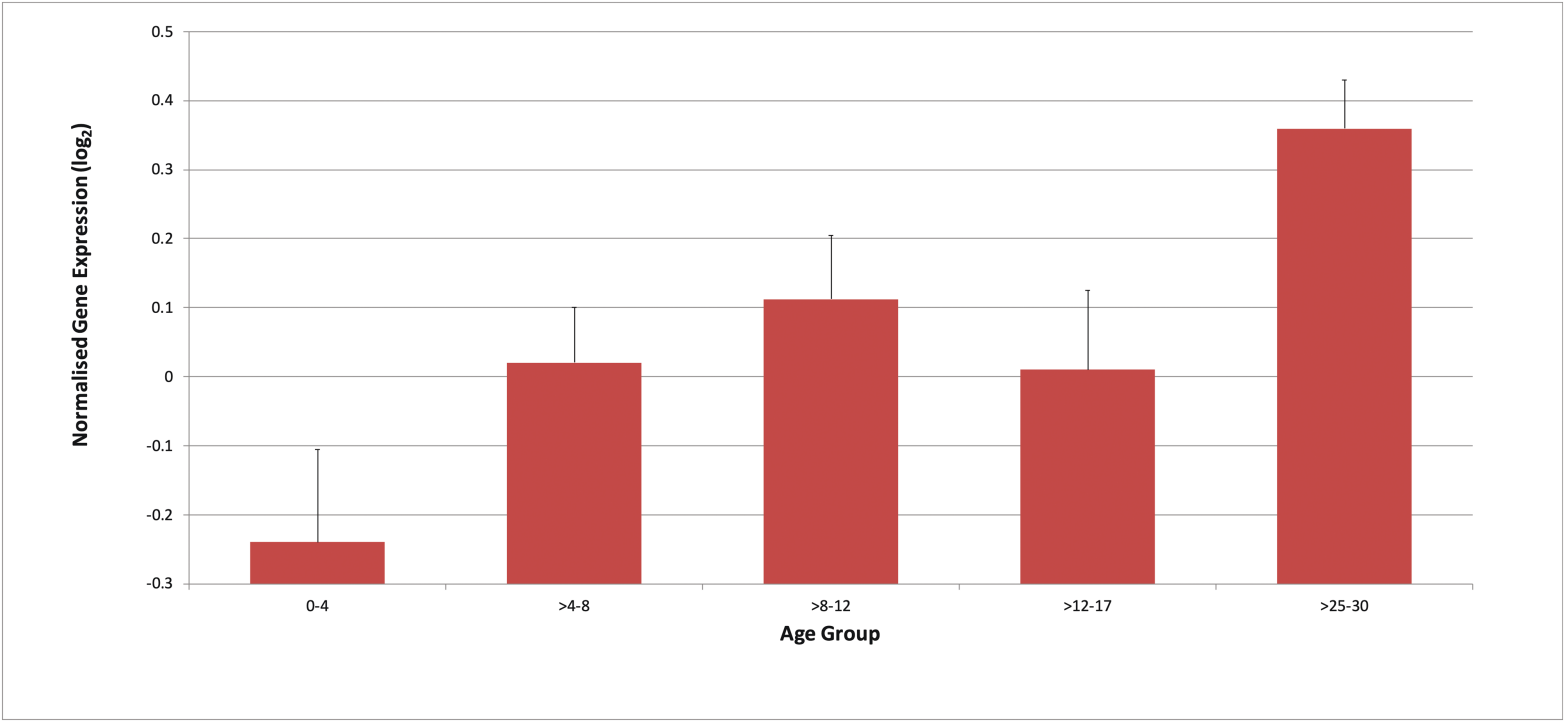
Expression of *ZBTB38* normalized for gender. Increasing age is associated with increased *ZBTB38* expression (*P* = 0.0007). Error bars represent SEM.

*ZBTB38* expression in healthy children demonstrated a positive correlation with age (see Fig. 1). Rank regression analysis identified a significant change in expression over time (*P* = 0.0007).

### Clinical and Endocrine Phenotype Associated With s6764769 and rs724016

#### EPIGROW study

Genotype was available for 261 patients for rs724016 and 210 patients for rs6764769. A comparison of the 2 SNPs indicated that they are in linkage disequilibrium with rs6764769 genotype sharing a 99.9% overlap with rs724016 where genotype data was available for both SNPs. Clinical phenotype data is therefore presented for rs724016 alone and is displayed in Table 1. For this SNP, the reference allele was A and variant allele was G; 128 patients were homozygous AA, 101 heterozygous AG, and 32 homozygous GG. Birth length was reduced in the homozygous GG genotype at −1.4 SD compared with −0.64 SD for homozygous AA genotype and −0.5 SD for the heterozygous group (*P* = 0.01). While there was a significantly lower peak growth hormone (GH) level on the first GH stimulation test in the homozygous GG group (*P* = 0.02), this was not present in the second test (*P* = 0.4). There were no significant differences in height standard deviation score (SDS), weight SDS, body mass index (BMI) SDS, sitting height SDS, head circumference SDS, fasting glucose, or fasting insulin.

**Table 1.** Clinical phenotype in the EPIGROW study associated with rs724016 genotype

Genotype	AA	AG	GG	*P* value
N (%)	128 (49)	101 (39)	32 (12)	
Male, n (%)	86 (66)	63 (62)	15 (47)	0.10
Age, years	8.7 ± 3.2	7.9 ± 3.1	8.5 3.3	0.16
Gestational age, weeks	38.5 ± 2.2	38.4 ± 3.0	37.7 ± 2.8	0.24
Birth length SDS	-0.6 ± 1.3	-0.5 ± 1.3	-1.4 ± 1.5	0.01
Birth weight SDS	-0.6 ± 1.4	-0.6 ± 1.1	-0.8 ± 1.0	0.73
Height SDS	-2.9 ± 0.7	-2.9 ± 0.7	-2.8 ± 0.5	0.79
Weight SDS	-2.5 ± 1.2	-2.6 ± 1.1	-2.5 ± 1.2	0.92
BMI SDS	-0.7 ± 1.3	-0.7 ± 1.1	-0.7 ± 1.1	0.98
Sitting height SDS	-2.5 (1.3)	-2.6 (1.0)	-2.7 (0.8)	0.79
Head circumference SDS	-2.2 ± 1.2	-2.0 ± 1.4	-1.8 ± 1.3	0.35
Fasting glucose (mmol/L)	4.6 ± 0.5	4.5 ± 0.4	4.4 ± 0.7	0.19
Fasting insulin (pmol/L)	25.2 ± 21.4	29.6 ± 24.8	20.9 ± 15.2	0.38
GH peak stimulation test (ng/mL)	14.7 (10.4)	12.0 (7.7)	12.3 (5.9)	0.004
IGF-I SDS	-2.1 ± 1.0	-1.9 ± 1.0	-1.8 ± 1.1	0.39

All data are given as mean ± SD except GH peak stimulation test and sitting height, which are median (interquartile range).

Abbreviations: BMI, body mass index; GH, growth hormone; IGF-I, insulin-like growth factor I; SDS, standard deviation score.

#### Growth study cohort

A total of 93 patients DNA were genotyped for rs724016, 40 patients were homozygous AA, 40 heterozygous AG, and 13 homozygous GG. There were 43 patients with growth hormone deficiency (GHD), 22 with Turner syndrome (TS), 12 were born small for gestational age, 7 had Prader-Willi syndrome, 5 ISS, and 4 had a skeletal dysplasia. Clinical phenotype data is included in Table 2. There was no difference between genotypes for 2 measures of response to GH therapy—height velocity SDS during the first year of therapy and change in height SDS during the first year of therapy (see Table 2). There were also no significant differences in any of the demographic variables (gender, age at start of treatment, birth weight SDS, as well as height, weight, and BMI SDS at start of treatment) (see Table 2). Although the homozygous GG group did have a lower height velocity SDS during the first year of treatment (1.4 ± 2.1 SD for GG vs 2.5 ± 2.6 SD for AG and 2.8 ± 3.2 SD for AA) and lower change in height SDS over the first year of treatment compared with the other groups (0.4 ± 0.3 SD for GG vs 0.7 ± 0.6 SD for AG and 0.7 ± 0.5 SD for AA), this did not reach significance.

**Table 2.** Clinical phenotype in the Child Growth Study associated with rs724016 genotype

Genotype	AA	AG	GG	*P* value
N (%)	40 (43)	40 (43)	13 (14)	
Male, n (%)	19 (47)	14 (35)	5 (38)	0.51
Age at start, years	7.0 ± 4.0	7.1 ± 3.6	6.6 ± 3.4	0.9
Birth weight SDS	-0.8 ± 1.4	-0.9 ± 1.6	-1.2 ± 1.0	0.71
Height SDS	-2.9 ± 1.5	-2.7 ± 1.3	-3.1 ± 1.2	0.54
Weight SDS	-1.9 ± 2.3	-1.6 ± 2.0	-2.2 ± 2.3	0.68
BMI SDS	0.3 ± 1.8	0.5 ± 1.5	-0.2 ± 2.2	0.50
Starting GH dose	28 ± 8.4	26 ± 6.9	27 ± 8.3	0.62
Change in height SDS over year 1 of treatment	0.7 ± 0.5	0.7 ± 0.6	0.4 ± 0.3	0.26
Height velocity SDS in year 1 of Treatment	2.8 ± 3.2	2.5 ± 2.6	1.4 ± 2.1	0.32
Change in height SDS over years 1 + 2 of treatment	1.1 ± 0.7	1.1 ± 1.1	0.9 ± 0.5	0.70
Height velocity SDS over years 1 + 2 of treatment	2.4 ± 2.6	2.1 ± 2.5	0.9 ± 2.0	0.20

All data are given as mean ± SD.

Abbreviations: BMI, body mass index; GH, growth hormone; SDS, standard deviation score.

We then modeled rs724016 with the variant G allele for both dominant (AA vs AG/GG) and recessive (AA/AG vs GG) modes of inheritance and compared the clinical data and response to GH therapy. There were no significant differences in gender, age, birth weight SDS, height SDS, weight SDS, or BMI SDS prior to GH treatment for either the dominant or recessive models (see Table 3). For the dominant mode of inheritance there was no evidence of any effect on height velocity or change in height SDS during the first 2 years of treatment. When the recessive model of inheritance was considered, there was a significantly lower change in height SDS over the first year or treatment in the GG group (AA/AG vs GG = 0.7 ± 0.6 SD vs 0.4 ± 0.3 SD, *P* = 0.02) with a trend toward lower height velocity SDS both in the first year of treatment (2.6 ± 2.9 SD vs 1.4 ± 2.1 SD, *P* = 0.08) and when combining the first and second years of treatment (2.2 ± 2.5 SD vs 0.9 ± 2.0 SD, *P* = 0.06).

**Table 3.** Clinical phenotype in the Child Growth Study associated with rs724016 genotype modeled by mode of inheritance of the rs724016 SNP

Modeled mode of inheritance	Dominant			Recessive		
Genotype	AA	AG/GG	*P* value	AA/AG	GG	*P* value
N (%)	40 (43)	53 (57)		80 (86)	13 (14)	
Male, n (%)	19 (47)	19 (36)	0.35	33 (41)	5 (38)	1.0
Age at start, years	7.0 ± 4.0	7.0 ± 3.6	0.92	7.1 ± 3.7	6.6 ± 3.4	0.64
Birth weight SDS	-0.8 ± 1.4	-0.9 ± 1.5	0.90	0.9 ± 1.5	-1.2 ± 1.0	0.33
Height SDS	-2.9 ± 1.5	-2.8 ± 1.2	0.80	-2.7 ± 1.3	-3.1 ± 1.2	0.30
Weight SDS	-1.9 ± 2.3	-1.8 ± 2.0	0.99	-1.7 ± 2.1	-2.2 ± 2.3	0.48
BMI SDS	0.3 ± 1.8	0.4 ± 1.6	0.98	0.4 ± 1.6	-0.2 ± 2.2	0.34
Starting GH dose	28 ± 8.4	26 ± 7.2	0.41	26± 7.9	27 ± 8.3	0.99
Change in height SDS over year 1 of treatment	0.7 ± 0.5	0.7 ± 0.6	0.75	0.7 ± 0.6	0.4 ± 0.3	0.02
Height velocity SDS in year 1 of treatment	2.8 ± 3.1	2.3 ± 2.7	0.47	2.6 ± 2.9	1.4 ± 2.1	0.08
Change in height SDS over years 1 + 2 of treatment	1.1 ± 0.7	1.1 ± 1.1	0.65	1.1 ± 1.0	0.9 ± 0.5	0.35
Height velocity SDS over years 1 + 2 of treatment	2.4 ± 2.6	2.0 ± 2.5	0.50	2.2 ± 2.5	0.9 ± 2.0	0.06

The variant allele is G. Assuming dominant inheritance we compared the AA genotype group to the AG and GG groups. For recessive inheritance we compared the GG group to the AA and AG groups.

Abbreviations: BMI, body mass index; GH, growth hormone; SDS, standard deviation score.

Recognizing that diagnosis has a profound impact on response to therapy, we divided the cohort into each diagnostic group (GHD, Turner syndrome, small for gestational age, Prader-Willi syndrome, ISS, and skeletal dysplasia). Using the dominant inheritance model, there were no significant effects demonstrated on change in height SDS or height velocity SDS either over year 1 or over year 1 and year 2 of treatment. Using the recessive model of inheritance, only the GHD and Turner syndrome groups had a sufficient number of individuals with GG genotype to attempt analysis (for GHD GG n = 5, AA/AG n = 37 and for Turner syndrome GG n = 6 AA/AG n = 16). For the GHD subjects there were no significant differences in height velocity SDS either in year 1 or years 1 and 2 of treatment and no significant difference in the change in height SDS over the first 2 years of treatment. There was, however, a lower change in height SDS over the first year of treatment in the GG group (GG vs AA/AG = 0.3 ± 0.4 SD vs 0.9 ± 0.7 SD, *P* = 0.02). No significant differences were found in the TS group when analyzed using the recessive modal on any of the growth hormone response parameters.

### siRNA-Mediated Knockdown of *ZBTB38*

**Figure 2.**
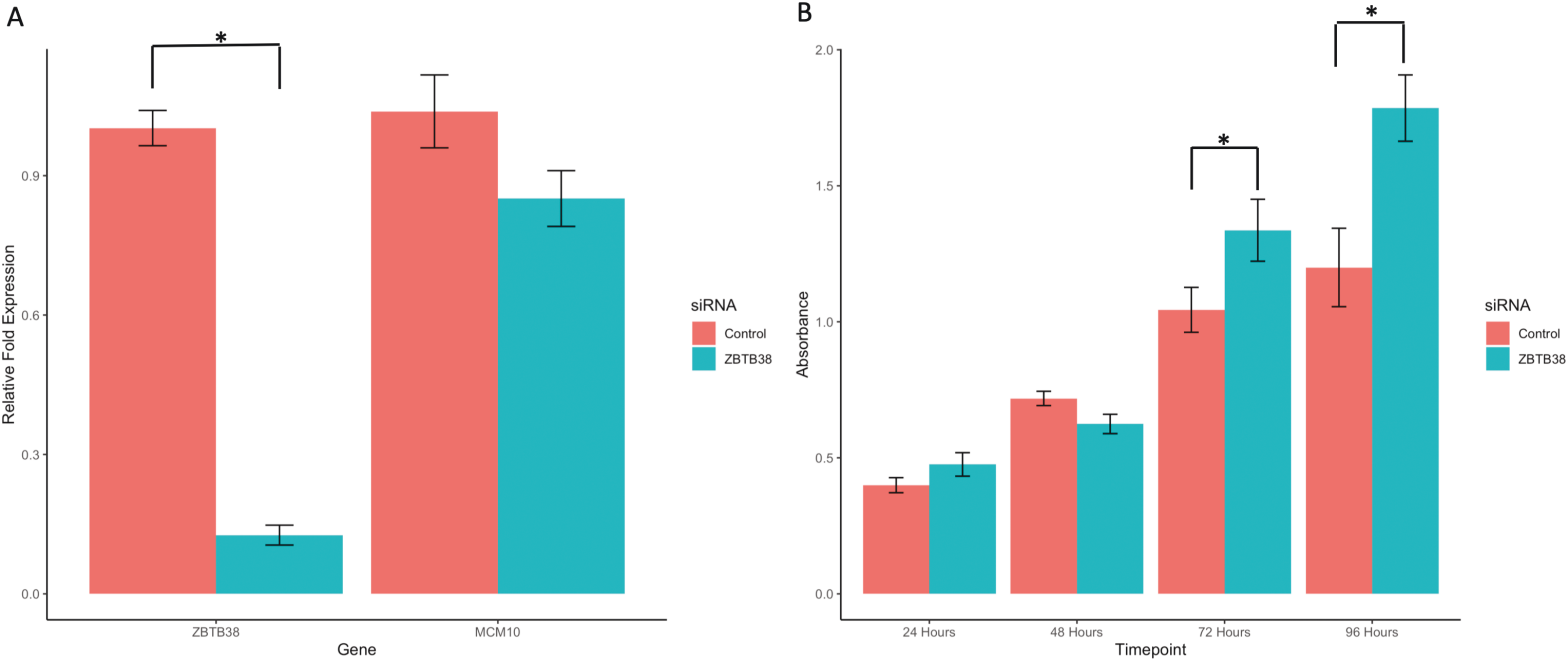
A, Relative fold gene expression for *ZBTB38* and *MCM10.* A siRNA targeted against *ZTB38* successfully reduced expression of *ZBTB38* but did not alter expression of *MCM10*. B, WST-8 cell growth assay demonstrated increased cell growth in the presence of *ZBTB38* knockdown at 72 and 96 hours posttransfection. *P < 0.05. Error bars represent SEM.

Relative fold gene expression of *ZBTB38* following treatment with 5nM *ZBTB38* siRNA was 0.12 ± 0.05 and 1.00 ± 0.09 in control siRNA treated cells (*P* < 0.001). Relative fold expression of *MCM10* in the *ZBTB38* siRNA knockdown cells was 0.85 ± 0.2 compared with 1.0 ± 0.27 in control siRNA treated cells (*P* = 0.07) (see Fig. 2A). Cell growth as measured by WST-8 assay was not different between the *ZBTB38* knockdown and control cells at 24 or 48 hours but was increased at 72 hours (*P* = 0.04) and 96 hours (*P* = 0.03) posttransfection in the *ZBTB38* siRNA knockdown cells (see Fig. 2B).

## Discussion

In the EPIGROW study, which compared cases of ISS to control subjects, 2 SNPs were identified as being associated with ISS: rs6764769 and rs724016. The first finding from our study is that both these SNPs are in linkage disequilibrium. For both of these SNPs the reference allele was A and variant allele G, with the variant G allele frequency known to be lower in cases than controls in the original EPIGROW study.

Previous studies have highlighted the role of *ZBTB38* SNPs in ISS, but the phenotype associated with these SNPs has not been explored. We found no difference on sitting height, head circumference, body mass index, weight, or height between the *ZBTB38* genotypes in a population of children with ISS. There was a significant decrease in birth length in the ISS population in the presence of a homozygous G allele. In the EPIGROW study, a homozygous G genotype was less frequently found in the ISS population compared with controls. This may indicate that the AA *ZBTB38* genotype affects postnatal more than antenatal growth given its association with ISS, and that the GG genotype is linked with lower birth length. There was a similar trend of the effect of GG genotype on birth size in our growth study cohort with birth weight SDS −1.2 in the GG group compared with −0.8 and −0.9 SD in the AA and AG groups, respectively, but this did not approach significance. The AA genotype patients had higher peak GH levels on the first GH stimulation test with insulin-like growth factor I (IGF-I) concentrations that were lower than the other groups (but this did not meet significance). This raises the possibility that the AA genotype may affect growth through a mild form of IGF-I resistance but the better response to recombinant human growth hormone (rhGH) therapy is less indicative of an IGF-I resistance phenotype.

Having examine the phenotype associated with the *ZBTB38* SNPs, we explored the influence of genotype on the response to treatment with rhGH. We found evidence that the response to treatment was better in the AA and AG patients combined compared to patients with a GG genotype with an improved change in height SDS over the first year of treatment. While the change in height SDS over 2 years of treatment was not significant, there was still a trend in favor of better response in the AA/AG group. One weakness of the short stature cohort used is that it included several different indications for GH, and it is well recognized that response to rhGH treatment varies with treatment indication (GHD, Turner syndrome, small for gestational age, etc). We therefore analyzed the cohort by individual indications for GH. The better response in the AA/AG genotypes for change in first-year height SDS persisted in the GHD subgroup. This was the largest subgroup of the cohort and the failure to find a significant effect on GH response by genotype in Turner syndrome may simply reflect the lower numbers in that group.

SNPs in *ZBTB38* have previously been associated with adult height within the normal population as well as with ISS. In the study by Gudbjartsson et al, the authors found that for one SNP in *ZBTB38* (rs6763931), the A genotype was positively correlated with both height and gene expression in blood and adipose tissue [6]. In our study, there was a positive correlation between age and *ZBTB38* expression in healthy control children. There is, however, another potential but perhaps more speculative interpretation of the expression data—that *ZBTB38* expression may be inversely related to growth rate. Longitudinal growth occurs by endochondral ossification and is highest during fetal life and infancy, falling thereafter to a slow rate through childhood before increasing once more during the pubertal growth spurt. We observed the lowest levels of *ZBTB38* gene expression during the first 4 years of life followed by higher levels during mid-childhood and a decrease in expression aged 12-17 years, which would be associated with the pubertal growth spurt. At the end of puberty, growth ceases and we observed an increase in *ZBTB38* gene expression. Our study is limited by the use of blood gene expression and small numbers of subjects in each age group; however, unlike in the data reported by Gudbjartsson, we measured gene expression during childhood when growth is actually occurring rather than in adulthood when growth has ceased. The limited number of patients in each age group does mean that we cannot prove a significant decrease in *ZBTB38* gene expression in the pubertal years, but the in vitro findings on gene knockdown leading to increased cellular growth would support this theory. Of note, if the association with growth rate is correct, then the age-related association would be a false result driven by the very low expression in the youngest (and fastest growing) subjects compared to the slow/no growth in the young adult group.

Our own data on the effects of siRNA knockdown of *ZBTB38* are consistent with the finding that *ZBTB38* expression inhibits growth as we observed an increase in cell proliferation with *ZBTB38* depletion. Other in vivo data on the effects of *ZBTB38* on growth are inconsistent with decreased expression seen in mouse models of muscle regeneration [18] but decreased proliferation seen with Cibz knockdown in mouse embryonic stem cells [19]. In prostate cancer *ZBTB38* expression is lower in tumors compared with benign prostatic hypertrophic and normal tissue, and lower expression is associated with higher pathological grade and poorer outcome [24]. Depletion of *ZBTB38* in prostate cancer cell lines, however, did not alter cell proliferation. ZBTB38 is known to be a key regulator of genomic stability via the replication protein MCM10 [20]. RBBP6 is responsible for the ubiquitination and proteasomal degradation of ZBTB38, while ZBTB38 is a transcriptional repressor of *MCM10*. Loss of RBBP6 therefore results in upregulation of ZBTB38 and suppression of *MCM10* transcription, which results in impaired genome stability and genome under replication [20]. One patient with a compound heterozygous loss-of-function mutation in *MCM10* has been described with natural killer cell deficiency and impaired cell proliferation [25]. The observation from our in vivo and in vitro study on the inverse relationship between *ZBTB38* expression and growth would fit with this model.

There is a complex interplay between the effects of ZBTB38 on cell proliferation and genome instability. Treatment of a variety of cancer cells with DNA methyltransferase inhibitors (DNMTi) results in downregulation of ZBTB38 protein expression via proteasomal degradation due to upregulation RBBP6 [21]. Marchal et al studied the effects of *ZBTB38* depletion via RNA interference on a variety of cancer cell lines and found no change in K562 or THP-1 cell proliferation, but an increase in proliferation in MOLM-14 cells [21]. In contrast, in the presence of DNMTi, *ZBTB38* expression strongly inhibited cell proliferation and increased cell death in all cell lines [21]. *ZBTB38* downregulation was associated with upregulation of CDKN1C mRNA and protein, and this upregulation is key to the sensitivity to DNMTi treatment [21]. Gain-of-function mutations in *CDKN1C* cause IMAGe and Silver-Russell syndrome, in which there is growth impairment [22]. The role of ZBTB38 in physiological and pathological growth is complex and there may well be tissue- and developmental stage–specific effects.

In conclusion, this study demonstrates an inverse relationship between peripheral blood mononuclear cell *ZBTB38* expression with normal growth and that the *ZBTB38* SNPs associated with ISS are linked to higher birth size and improved response to rhGH therapy.

## Data Availability

Restrictions apply to the availability of some or all data generated or analyzed during this study to preserve patient confidentiality or because they were used under license. The corresponding author will on request detail the restrictions and any conditions under which access to some data may be provided.

## Abbreviations

BMI: body mass index
DNMTi: DNA methyltransferase inhibitors
GH: growth hormone
GWAS: genome-wide association studies
IGF-I: insulin-like growth factor I
ISS: idiopathic short stature
PCR: polymerase chain reaction
rhGH: recombinant human growth hormone
SDS: standard deviation score
siRNA: small interfering RNA
SNP: single-nucleotide polymorphism
ZBTB38: Zinc-finger and BTB Domain Containing 38
